# Development of the accredited duchenne centers program, a global program to achieve uniform and up-to-date care for all people living with duchenne muscular dystrophy

**DOI:** 10.1186/s13023-025-03843-9

**Published:** 2025-07-02

**Authors:** Imelda J. M. de Groot, Karolina Podolská, Nathalie Goemans, Suzie-Ann Bakker, Nawel Lalout, Merel Jansen, Elizabeth Vroom

**Affiliations:** 1 World Duchenne Organization, Landjuweel 34, 3905 PG Veenendaal, The Netherlands; 2 Accredited Duchenne Centers Advisory Board, Veenendaal, The Netherlands; 3 Duchenne Parent Project, Veenendaal, The Netherlands

**Keywords:** Accredited duchenne centers (ADC) program, Duchenne muscular dystrophy, Certification, Global, Care considerations, Consensus guidelines

## Abstract

The Accredited Duchenne Centers (ADC) Program is a global program initiated by the World Duchenne Organization (WDO). The aim of the ADC Program is to achieve global, uniform and up-to-date care for all people living with Duchenne Muscular Dystrophy (DMD) that are in line with the latest international care considerations and care guidelines. This will be realized by accrediting Duchenne care centers for children and/or adults that provide comprehensive care according to the latest care considerations, and supporting care centers that do not yet meet these criteria for accreditation, to improve care provided to DMD patients. The accreditation process consists of five stages ranging from application to maintenance of accreditation by continuous education. The accreditation criteria and procedures were developed in close collaboration with an international Advisory Board. This paper describes the development and content of the ADC Program. The ADC Program is the first global accreditation program for both children and adults with DMD. The results of the program may contribute to optimal implementation of the latest research insights and optimum care for all people living with DMD and their families.

## Article highlights

- Global accreditation program for Duchenne Muscular Dystrophy: the Accredited Duchenne Centers (ADC) Program
- Care provided according to the care considerations, consensus guidelines and expert opinions
- Professionals, patients and families are involved in the development of the ADC Program
- Continuous education is part of the program
- The ADC Program contributes to global, uniform and up-to-date care for all people living with Duchenne muscular dystrophy
- A FAIR database stores interview recordings and questionnaire results to provide transparency and identify barriers to meeting specific criteria

## Introduction

Good psychosocial and physical care has a positive influence on life satisfaction and life expectancy in many diseases, including Duchenne Muscular Dystrophy [1–7]. Duchenne Muscular Dystrophy (DMD) is the most common genetic neuromuscular disorder in children. The pooled global DMD birth prevalence is 19.8 (95% CI:16.6–23.6) per 100,000 live male births [8]. The absence, or near absence, of the protein dystrophin results in a steady loss of vulnerable muscle fibers together with a loss of muscle mass and muscle strength. Patients, mainly boys and men because of its x-linked chromosomal recessive character, become wheelchair-dependent in their early teenage years and subsequently lose their arm functions. Although there are promising treatments that can delay progression, many of these are still in a research stage. This means that patients have to rely on optimal preventative and symptomatic care that may extend their life expectancy to over 30 years of age with good quality of life [7–11]. With increased life expectancy, there is now a significant group of adult patients with DMD. Adult patients with DMD have their own specific challenges, requiring a different and more multisystemic organization of healthcare than children [12].

International care considerations for children with DMD [13–15] and additional guidelines for adult patients with DMD have been published [16, 17]. The aim of the care considerations and guidelines is to standardize care for everyone living with DMD and to reach optimal quality of life and life expectancy. Care considerations include 11 topics: considerations for diagnosis, neuromuscular rehabilitation, endocrine, gastrointestinal, respiratory, cardiac, bone health, orthopedic, emergency, psychosocial management, and transition of care across the lifespan. Consensus guidelines for adults describe the focus of care shifting from a preventive towards a treatment approach. Therapy guidelines for adults assist therapists in their clinical practice. Although care considerations and consensus guidelines are freely available online, it is well known that compliance is suboptimal due to multiple reasons [18, 19]. Disregarding the care considerations increases the risk of preventable medical complications and differences in quality of life and life expectancy between care centers and individual patients.

Currently, there are no international agreements on the implementation of the care considerations and guidelines for DMD. Care centers can make their own choice to follow the considerations or not, which increases the risk of considerable differences in the care provided, together with all the associated risks. Patients and families may not be aware of differences in quality of care and are often in a position of being dependent on their care provider. An accreditation or certification program can help overcome this problem. Accredited Duchenne care centers recognize the need for and provide up-to-date, high quality and uniform care. In the United States, Parent Project Muscular Dystrophy (PPMD) has developed the Certified Duchenne Care Center Program [20], but a worldwide applicable accreditation program was lacking. For this reason, the World Duchenne Organization initiated the global Accredited Duchenne Centers (ADC) Program. The aim of the ADC Program is to achieve global, uniform and up-to-date care for all people living with DMD. To stay up to date means also that continuous education is part of the program. Duchenne care centers can obtain accreditation if they provide care according to the international care considerations, consensus guidelines and/or expert opinions. Both healthcare professionals and patients and families are involved in the development of the ADC Program. The aim of this paper is to describe the development and content of the ADC Program.

## Methods

### Design

This paper describes the developmental process and content of the Accredited Duchenne Centers (ADC) Program. The program started in spring 2022 with the preparation phase, in which a program team was formed and the Advisory Board was set up. A definition of accreditation was established (phase 0). Meanwhile, accreditation criteria have been defined based on the international consensus statements, guidelines and expert opinions. Subsequently, pre-visit questionnaires and visitation interviews were developed (phase 1). Also in phase 1, building a database was begun. During the pilot phase (phase 2), there was a call for applications and Duchenne care centers were recruited to participate. The last phase (phase 3) is the implementation of the program worldwide.

### Preparation phase – phase 0

#### Starting the process

The Accredited Duchenne Centers (ADC) Program was initiated and is financed by the World Duchenne Organization (WDO). WDO approached the first author, and together with the WDO a program team was established. The ADC Program team consists of the authors of this article.

### Assembling the advisory board

Members of the Advisory Board are all professionals with expertise in the field of DMD. Members were recruited via the WDO network. Next to professionals, patient representatives were asked to join the Advisory Board to guarantee the input of patients from the beginning of the program. The international Advisory Board consisted initially of 11 members with a multidisciplinary background from 8 different countries, and is chaired by Prof. Nathalie Goemans. Recently a 12th member has joined the advisory board (see here for members https://accreditedduchenne.center).

## Results

### Phase 1: defining accreditation

The ADC Program team and Advisory Board reached consensus on the following definition for accreditation for children and/or adults: “Accredited Duchenne Centers for children and/or adults provide care according to criteria that are in agreement with the latest care considerations and/or expert opinions for children and/or adults. Care is provided by a multidisciplinary team and may be delivered either by a single center, or shared with other locations; however, the applying center is responsible for the care plan, coordination, communication, documentation and collaboration. Although the definition is comparable for both children and adults, the care considerations or consensus statements on which the criteria are based differ.

### Defining accreditation criteria (and a minimum set for visitation)

A structured process consisting of a literature review by the ADC Program team resulted in a first concept of accreditation criteria for children and adults. Accreditation criteria were primarily based on the latest care considerations and consensus and therapy guidelines for children and adults. Accreditation criteria were reviewed and discussed with the Advisory Board. The first set of accreditation criteria was adjusted based on feedback from the Advisory Board. The current set of accreditation criteria for children and adults for which agreement was reached can be found in Appendix 1 and 2. A minimum set of accreditation criteria was determined and has to be achieved before a visitation can be planned (these are mentioned in Appendix 1 and 2 as SCREENING).

### Developing the accreditation procedure

The accreditation procedure was developed by the ADC Program team and the Advisory Board. The process consists of five steps (step 1 Registration, step 2 Screening, step 3 Visitation, step 4 Accreditation, step 5 Maintaining accreditation) which will be further explained in the results section.

#### Step 1 registration

Centers that apply for accreditation need to start by registering. This can be done via the ADC-website (https://accreditedduchenne.center). Centers complete and submit their registration form on the ADC website. Centers can apply for accreditation for children or adults or for both.

#### Step 2 screening

After registration, Duchenne centers receive a link to a digital pre-visitation questionnaire to determine whether they provide care according to the care considerations, and/or consensus and therapy guidelines. There are two separate questionnaires, one for pediatric care and one for adult care. Together with the questionnaire(s) centers will be asked to submit a letter of support from the national patient organization. The completed questionnaire(s) and a letter of support are both required prior to a visitation. After assessment of the pre-visitation questionnaire and possible additional questions from the ADC Program team, provided the center fulfilled the pre-visitation criteria, a visitation to the center is planned.

#### Step 3 visitation

A visitation is performed by a visitation committee consisting of a group of experienced professionals and a patient representative. (Semi) structured interviews, based on all criteria, are conducted with individual professionals (physicians, therapists, psychologists, social workers, nurses) and patients and their families. Interviews per topic of management take approximately 30–90 min. A visitation takes two days if care is given either to pediatric or adult patients. Visitation of a center with a pediatric and an adult team that applies for both pediatric and adult accreditation requires 2–3 days, depending on whether there is one team for both patient groups or two separate teams.

In addition, the national supporting patient organization is asked to send a questionnaire to members who receive care in the applying center. The questionnaires sent to patients receiving care in the applying center are closed questions based on the criteria combined with open questions to allow for specific comments and voicing preferred changes in care management. Their answers and the answers given in the interviews of the patients and their families during the visitation are compared to the answers provided by the professionals on several topics.

Interviews with professionals are recorded and stored according to the FAIR data principles (see results) for which the professionals signed informed consent.

#### Step 4 accreditation

Interviews are reviewed by the visitation committee, and in case of doubt the Advisory Board is asked for their advice. If criteria for accreditation are met, centers become an Accredited Duchenne Center for pediatric and/or adult care. Centers providing most care according to the care considerations, but not meeting all criteria for accreditation yet, receive advice to make some (minor) changes to the care provided based on written feedback by the ADC Project Team. Follow-up contact by email or an online meeting is planned when these centers have processed the changes. Centers can then obtain their accreditation status.

#### Step 5 maintaining accreditation

Accreditation will be maintained for five years (unless major changes in health care organization and/or the care provided are made, which the centers are obliged to report). During these five years Accredited Duchenne Centers will need to follow the ongoing education program provided by the WDO in order to remain up-to-date. An online re-accreditation procedure will be scheduled after five years (or earlier if necessary). If needed a new visitation will take place.

### Database

To enable transparency of the whole process of accreditation, a FAIR database was set up [21, 22]. FAIR means Findable, Accessible, Interoperable and Reusable. With the help of Mark Wilkinson (*BBVA-UPM Industry Chair on Biotechnology, Co-Founder of FAIR Data Systems, Spain)* the FAIR database was constructed and the results of the pre-visitation questionnaires and the patients’ questionnaires are directly entered in the database. The FAIR database is queryable as both questions and answers are coded with unique identifiers and transformed into machine-language format, using the Resource Description Framework Standards (W3C Semantic Web Standards, Resource description framework (RDF) https://www.w3.org/RDF/). This makes it distinct from a standard database, which is a long-term document/file repository. It is therefore “ready” to be integrated in the future with third-party data. It is the intention to store the results of the interviews in the database as well. The aim of this database is to gain insight into the barriers and issues to meeting specific criteria and to focus support and education on these topics. An additional database was set up where raw data can be stored, such as the recordings of the interviews. This can be used to listen back in case of confusion or dispute.

### Pilot phase–phase 2

Centers that care for persons with DMD were invited to apply for accreditation in February 2023. Interested centers submitted their registration on the ADC website. Several centers applied and 6 centers were selected that fulfilled the visitation criteria based on the pre-visitation questionnaire and were supported by a national patient organization. Three visitations were performed, and after minor changes in care management, all 3 were accredited. After the pilot phase, interviews with the patients and their families were adjusted to a more open question interview. Patient interviews and questionnaires gave good insight into the perspective of patients and their families regarding the care provided by the applying center. These are compared with the answers given by the professionals and can potentially be discussed with the professionals.

Two fully prepared visitations had to be canceled due to reasons beyond our control; one visitation was canceled by the applying center. This experience has changed our procedure regarding visitations: we now ask the applying center to confirm the visitation dates and if they cancel they have to pay a fee to cover any costs already incurred by the WDO.

## Discussion

The aim of this Accredited Duchenne Centers Program is to attain worldwide uniform and up to date optimal care for persons with Duchenne Muscular Dystrophy. For this reason, criteria were formulated based on the standards of care, care considerations and consensus statements. All applying centers will be judged in accordance with these criteria independent of the country and health system they are working in the Certified Duchenne Care Center Program developed in the USA [20] is highly comparable as it is based on the same care considerations. However, this program is focused on the situation in the USA and not every country has the same health organization. We tried to formulate our criteria to be applicable worldwide, as there are several ways to fulfill a criterion. For example, wheelchair prescriptions can be written by occupational therapists or physiotherapists or by a special aids team etc. To reach the criterion attention, must be given to seating position, the ability to still perform activities, sitting without pain and adaptability.

The benefits of the whole process to reach accreditation are a full screening of the team and the care program of the applying center. Suggestions to improve are made and new insights can be gained based on the experience of how other centers provide care. During the application process, the pre-visitation questionnaires and interviews already enable centers to make changes that are supported by the hospital in which they are centered. It gives the patient organization(s) in that country confidence to refer patients and parents to a center working according to the latest care considerations and guidelines. Ongoing education also helps the centers stay up to date. The aim is to build a network of accredited centers that can request advice and learn from each other. The possibility of becoming a member of a visitation team if your center is already accredited will also be offered; this will help to spread knowledge around the world.

A FAIR data system is in place to guarantee a transparent procedure. These are some of the advantages of having a FAIR database:, 1) Records deposited in a ‘container’ are assigned a globally unique persistent identifier (PID) 2) Access to datasets are controlled and include a license of use 3) Metadata remain available over time, even if the dataset is no longer available and 4) Metadata describing the datasets will use controlled vocabularies. Keeping the ADC datasets FAIR over time requires professional data curation and digital preservation [21]. The collected data will be analyzed to identify which criteria are hard to meet for most centers. These results will inform management of the education program. The World Duchenne Organization (WDO) has a yearly Duchenne Care Conference and this year (2024) one of the topics was based on the results of the ADC Program. Specific topics can be addressed through webinars. The attendance of a team at the follow- up education program will be registered.

After discussion with the Advisory Board on the timelines required to update and eventually adjust the criteria, it was agreed to revise the criteria every 5 years. If, however, new guidelines are published or new insights on a specific care field require major changes in care, the criteria will be adjusted sooner and the educational program will focus on these aspects.

Currently the program is being implemented worldwide (phase 3), with a number of visitations performed (N = 10) and planned (N = 1).

The best conclusion is a quote from one of the centers that has been visited and accredited: ´It has been a great privilege and honor to be selected for a visit as part of this ADC Program. We are a passionate team, and the accreditation process has confirmed the standards of care we set and excel in as a team and has also helped identify areas for improvement which we are committed to addressing, with the ultimate goal of trying to achieve excellence for our patients.”

## Consent for publication

All authors gave consent for publication.

## Conflict of interest

No authors have any conflict of interest. ADC Program is part of the World Duchenne Organization. The ADC Advisory Board has no conflict of interest.

## Data Availability

The database is FAIR, this means the data are Findable, Accessible, Interoperable and Reusable. ijmdg, mj, kp, sab, ng and ev set up the ADC program, nl is responsible for the FAIR database.

## Appendix 1

### Criteria for accreditation—children (aged 0–18 years)

Centers that apply for accreditation for children and meet the criteria can become an Accredited Duchenne Care Center for Children. Criteria for children are in agreement with the latest Standards of Care (SoC), consensus guidelines and/or expert opinions.^1,2,3^

Criteria for Accreditation for children are (SCREENING means that this criterion is also used in the pre-visitation questionnaire as well as in the visitation interviews):

### Organization of health care

- The number of children with DMD treated is in relation to the care region (SCREENING)
- Pediatric DMD care is provided by a multidisciplinary team (at least: neuromuscular specialist (rehabilitation physician and/or neurologist), cardiologist, respiratory physician, physiotherapist, occupational therapist, speech and language therapist, dietitian, clinical neuropsychologist) (SCREENING) with experience and expertise
- Multidisciplinary pediatric DMD care may be delivered either by one center, or shared with other locations; however, the applying center is responsible for the care plan, communication, documentation and collaboration (SCREENING)
- Multidisciplinary pediatric DMD care leads to an integrated care plan
- There is a DNA (Did Not Attend) policy for pediatric DMD patients (DMD patients are vulnerable and should not be discharged if they fail to attend appointments)

### Diagnosis

Genetic testing is used to confirm the diagnosis (SCREENING)

### Carriers and genetic counseling

- Family members at risk receive genetic counseling (SCREENING)
- Carrier testing is recommended for female relatives of boys with DMD
- Female carriers receive regular physical assessment (SCREENING)
- Female carriers have access to cardiac check-up (SCREENING)
- Female carriers receive a baseline cardiac assessment in early adulthood including electrocardiogram and non-invasive imaging (preferably cMRI)
- Female carriers receive cardiac check-up every 3–5 years
- Genetic counseling and physical assessments are tailored to mothers and siblings

### Neuromuscular management

- Patients are advised to use corticosteroids (SCREENING) from the early ambulatory stage onwards
- Annual education on adrenal suppression and stress dosing during illness

### Rehabilitation management

- Pediatric DMD patients have contact with the rehabilitation specialist at least every 6 months throughout life from diagnosis onwards, with more frequent appointments if needed
- Comprehensive multidisciplinary rehabilitation assessments are done at least once every 6 months in line with the disease stage (can be in shared care; assessments are done at least once per year in the main center) (SCREENING)
- Comprehensive multidisciplinary assessments, including standardized assessments, are provided across all domains of the International Classification of Functioning, Disability and Health (ICF) (including measures of passive range of motion, muscle extensibility, posture and alignment, strength, function, quality of everyday life)
- Multidisciplinary (anticipatory) interventions are provided across all domains of the ICF and contribute to:

Ambulatory children:

- Prevention of contractures or deformity, overexertion and falls;
- Promotion of energy conservation and appropriate exercise or activity;
- Provision of orthosis and equipment

Additionally, in non-ambulatory children:

- Provision of mobility devices, seating and support, standing devices, assistive technology and adequate housing;
- Support in pain and fracture prevention, applications for funding, participation, and self-determination on transition into adulthood
- Care (including physiotherapy and occupational therapy) is provided based on the assessments and individual needs of the patient

### Endocrine management

- Assessment of height is done at least once every 6 months (SCREENING) in ambulatory (standing height and non-standing height) and non-ambulatory (non-standing height) children
- Annual assessment of pubertal status (consider starting at age 12–13)
- Consider treatment with testosterone from age 13–14 years (if needed)
- Family education on adrenal crisis and stress dose steroid prescription if children are on glucocorticoids (SCREENING)

### Gastrointestinal and nutritional management

- Assessment by a nutrition dietitian at least annually (SCREENING)
- Assessment of nutritional status at least annually
- Assessment of body weight at least annually (SCREENING)
- Preventing both overweight and underweight (SCREENING), involving a dietitian and speech- and language therapist
- Annual assessment of the level of vitamin D (SCREENING)
- Annual assessment of dietary calcium intake (SCREENING)
- Treatment with a vitamin D supplement to maintain level > 30 ng/ML
- Treatment with calcium when dietary calcium intake is inadequate
- Gastrointestinal complications (including constipation, bloating, reflux, gastroparesis and swallowing dysfunction) are monitored every 6 months
- Gastrointestinal complications (including constipation, bloating, reflux, gastroparesis and swallowing dysfunction) are actively treated and prevented
- Annual discussion of gastrostomy tube feeding and timely intervention as part of usual care from the late ambulatory stage onwards

### Respiratory management

- Respiratory assessments are initiated in the ambulatory stage (SCREENING) at age 5–6 years’ old
- Respiratory function is measured if needed in ambulatory children
- Respiratory function is measured at least once every six months in non-ambulatory children (SCREENING)
- Respiratory assessments include forced vital capacity (FVC), maximum inspiratory and expiratory pressures (MIP and MEP), peak cough flow (PCF) and blood oxygen saturation by pulse oximetry (SpO_2_) for non-ambulatory children
- Children with DMD receive lung volume recruitment if needed (SCREENING); when FVC is 60% predicted or less (achieved with a self-inflating manual ventilation bag or mechanical insufflation-exsufflation device once or twice a day)
- Children with DMD start assisted coughing when FVC is less than 50% predicted, when PCF is < 270L/min, or when MIP is less than 60 cm H2O
- Children with DMD receive nocturnal ventilation if needed (SCREENING)
- Children with DMD are advised to receive up to date immunization according to the national guidelines (influenza vaccines, covid and pneumococcal vaccines) (SCREENING)

### Cardiac management

- Cardiac function is assessed at least once per year in children with DMD from diagnosis (more often if needed) (SCREENING)
- Cardiac function is assessed with an echocardiogram and/or electrocardiogram (SCREENING) at least once every year (more often if needed)
- Children with DMD receive a cMRI once every 2–5 years or on clinical indication
- ACE inhibitors or angiotensin receptor blockers are initiated by age 10 years (SCREENING) or earlier if there are cardiac abnormalities
- Eplerenone is initiated in children with evidence of myocardial fibrosis on MRI
- Standard heart failure interventions are provided with deterioration of systolic function (SCREENING)
- Annual monitoring of renal function in children on ACE inhibitors and/or diuretics

### Bone health management

- Lateral spine X-rays are performed at least once every 1–2 years in children with DMD on glucocorticosteroids to detect early manifestations of bone fragility (SCREENING)
- Lateral spine X-rays are performed at least once every 2–3 years in children with DMD not on glucocorticosteroids to detect early manifestations of bone fragility (SCREENNG)
- Consult with a bone health specialist at the earliest sign of a fracture
- Annual assessment of vitamin D concentration and dietary calcium intake
- Treatment with vitamin D supplement and/or calcium when vitamin D concentration decreases to less than 30 ng/mL or calcium intake is less than the recommended intake for age

### Orthopedic management

- Joint range of motion is assessed at least once every six months (SCREENING)
- Annual visual monitoring for scoliosis in ambulant children with DMD (SCREENING), with a spine X-ray if a spinal curve is observed or if a visual inspection alone is inadequate (such as in children with obesity)
- Visual monitoring for scoliosis at least once every six months in non-ambulatory children with DMD (SCREENING)
- In the early non-ambulatory stage:
- a spine X-ray is recommended when a child first becomes non-ambulatory (especially for less experienced clinicians who may have difficulties in visually monitoring the spine for curves), or if a visual inspection alone is inadequate (such as in children with obesity)
- once a curve has been detected with radiography, a spine X-ray is performed once every six months in skeletally immature individuals in the early non-ambulatory stage, and annually in skeletally mature individuals
- In the late non-ambulatory stage, a spine X-ray is performed annually when there is any concern about progression in individuals with a known scoliosis
- Consider surgery on foot and Achilles tendon in selected situations
- Consider intervention with spinal fusion in defined situations

### Psychosocial management

- A mental health clinician (such as a psychologist or social worker) is part of the multidisciplinary team (SCREENING)
- Mental health and quality of life of children with DMD and their families are assessed at every clinic (SCREENING)
- Children with DMD and their families receive ongoing mental support (SCREENING)
- Referral to a clinical neuropsychologist when concerns about developmental progress arise (can be considered at diagnosis)
- Neuropsychological evaluations and interventions for learning, emotional and behavioral problems are provided (SCREENING)
- Educational needs and available resources are monitored annually (each child has an educational plan)
- Age-appropriate independence and social development are encouraged

### Transition

- Active approach to transition (SCREENING)
- Engagement in (optimistic) discussion about future (SCREENING)
- Foster goal setting and future expectations for adult life
- Transition preparation should begin at 12 years of age by making patients and families aware of plans for health care transition
- Initiation of transition discussions and planning about health care, education and adult living (including encouraging autonomy by stimulating exploring options for independent living, transportation and activities of daily living) by age 13–14 years
- There is a care coordinator or social worker responsible for transition
- Monitoring progress of transition planning at least once per year during puberty
- Providing transition support and anticipatory guidance about health changes during puberty
- A transition plan is set in collaboration with an adult DMD center working closely with the adult service

### Primary care

The primary care provider is part of the care team and works in partnership with the multidisciplinary team

### Perioperative and anesthetic care

- Anesthetic care is preferably provided by centers with expertise in the care of DMD patients (this may not always be possible, for example in case of an emergency)
- In case of an emergency setting where transfer to the (expert) center is not possible, advice is given to the local hospital (involved) by the expert center (if the expert center knows that a patient is hospitalized in a local hospital; patients are educated by the expert center to inform professionals in local hospitals to get in contact with the expert centers)
- There is always a pre-anesthetic assessment which includes assessments of 1) airway (mouth opening), 2) cardiac status (echo/cMRI) and 3) respiratory status (SCREENING)
- There is close liaison between the surgical, anesthetic, cardiac and respiratory teams
- The anesthetists are aware that DMD patients are at risk of cardiac and respiratory decompensation and steroid induced adrenal insufficiency during and after surgery
- The anesthetist is aware of the risk of acute rhabdomyolysis when using halothanes

### Emergency management

- An emergency card is recommended for DMD patients
- In case of an emergency setting where transfer to the (expert) center is not possible, advice is given to the local hospital (involved) by the expert center (if the expert center knows that a patient is hospitalized in a local hospital; patients are educated by the expert center to inform professionals in local hospitals to get in contact with the expert centers)
- Pediatric DMD patients have a plan to manage steroid-induced adrenal insufficiency during acute illness

### Patient satisfaction

- Monitoring patient satisfaction with the health care services regularly

### References

Birnkrant et al. (2018). Diagnosis and management of Duchenne muscular dystrophy, part 1: diagnosis, and neuromuscular, rehabilitation, endocrine, and gastrointenstinal and nutritional management. Lancet Neurology; 17(3):251-267.

Birnkrant et al. (2018). Diagnosis and management of Duchenne muscular dystrophy, part 2: respiratory, cardiac, bone health, and orthopaedic management. Lancet Neurology; 17(4):347-361.

Birnkrant et al. (2018). Diagnosis and management of Duchenne muscular dystrophy, part 3: primary care, emergency management, psychosocial care, and transitions of care across lifespan. Lancet Neurology; 17(5): 445-455.

## Appendix 2

Accredited Duchenne Centers Program

Criteria for Accreditation – adults (aged ≥ 18 years).

Centers that apply for accreditation for adults and meet the criteria can become an Accredited Duchenne Care Center for Adults. Criteria for adults are in agreement with the latest Standards of Care (SoC), consensus guidelines and/or expert opinions**.**^1,2,3,4,5^

Criteria for Accreditation for adults are (SCREENING means that this criterion is also used in the pre-visitation questionnaire, next to the use in the visitation interviews):

### Organization of health care

- The number of adults with DMD treated is in relation to the care region (SCREENING)
- Adult DMD care is provided by a multidisciplinary team (at least: neuromuscular specialist (rehabilitation physician and/or neurologist), cardiologist, respiratory physician, physiotherapist, occupational therapist, speech and language therapist, dietitian, gastroenterologist, clinical neuropsychologist) (SCREENING) with experience and expertise
- Multidisciplinary adult DMD care may be delivered either by one center, or shared with other locations; however, the applying center is responsible for the care plan, communication and collaboration (SCREENING)
- Multidisciplinary adult DMD care leads to an integrated care plan
- Patient shared decision making
- There is a DNA (Did Not Attend) policy for adult DMD patients (adult DMD patients are vulnerable and should not be discharged if they fail to attend appointments)

### Neuromuscular management

- Continuation of corticosteroids is advised if evidence of benefit outweighs risk in adult DMD patients (SCREENING)
- Adult patients are warned about potential side effects of corticosteroids (including annual education on adrenal suppression and stress dosing during illness)
- All adult DMD patients have an emergency plan to manage adrenal insufficiency during acute illness
- Regular assessment of side effects of corticosteroids (SCREENING) (including: optician assessment, blood pressure, HBA1C, lateral spine X Ray, mood, skin, serum 25OH Vitamin D)

### Rehabilitation management

- Adult DMD patients have contact with the rehabilitation specialist at least every 6 months, with more frequent appointments if needed
- Comprehensive multidisciplinary rehabilitation assessments are done at least once every 6 months in line with the disease stage (can be in shared care; assessments are done at least once per year in the main center) (SCREENING)
- Comprehensive multidisciplinary assessments, including standardized assessments, are provided across all domains of the International Classification of Functioning, Disability and Health (ICF) (including measures of passive range of motion, muscle extensibility, posture and alignment, strength, function, quality of everyday life)
- Multidisciplinary (anticipatory) interventions are provided across all domains of the ICF and contribute to:
- Prevention of contractures or deformity, overexertion and falls;
- Promotion of energy conservation and appropriate exercise or activity;
- Provision of orthosis and equipment;
- Provision of mobility and seating devices, assistive technology and information about adequate housing;
- Support in pain and fracture prevention, applications for funding, participation, and self-determination in the transition to adulthood
- Care (including physiotherapy and occupational therapy) is provided based on the assessments and individual needs of the patient

### Physiotherapy

- Physiotherapy should be available on a long-term continuing basis to adults with DMD according to their need
- A physiotherapist reviews support and advice on ventilation and airway clearance to both prevent and treat chest infections at least annually
- Physiotherapists provide a contracture management plan to adult DMD patients, including 1) the recommendation to perform stretching exercises, 2) paying attention to 24-h positioning, 3) considering ankle–foot orthoses (AFO’s) to maintain ankle mobility, and 4) considering resting hand splints to patients with tight long digit flexors to maintain functional hand position
- Adult DMD patients’ posture is managed with a 24-h approach involving the assessment and management of all positions that a patient uses (i.e. lying, sitting, standing) (overlapping physiotherapist/occupational therapist)
- Adult DMD patients should have access to an adequately adapted wheelchair
- Physiotherapists encourage physical activity and promote maintenance of whole-body fitness

### Occupational therapy

- Assessments of function, performance and participation are performed to identify issues affecting participation in activities of daily living
- Adult DMD patients are encouraged to be physically and socially active (educational opportunities, voluntary or paid work)
- Adult DMD patients are encouraged to participate in leisure activities

### Speech and language therapy

- A speech and language therapist is part of the multidisciplinary team
- Swallowing and nutritional status are assessed yearly
- Recommendations concerning textures of food are provided
- Interventions for communication problems are proposed

### Endocrine management

- Education about adrenal crisis and stress dose steroid prescription if adults are on glucocorticoids (SCREENING)
- Endocrine blood tests should be considered where appropriate: testosterone level, thyroid function and synacthen test

### Gastrointestinal and nutritional management

- Assessment by a dietitian at least annually (SCREENING)
- Assessment of nutritional status at least annually
- Assessment of body weight at least annually (SCREENING)
- Preventing both overweight and cachexia (SCREENING), involving a dietitian and speech- and language therapist
- Annual assessment of the level of vitamin D (SCREENING)
- Annual assessment of dietary calcium intake (SCREENING)
- Treatment with a vitamin D supplement to maintain level > 30 ng/ML
- Treatment with calcium when dietary calcium intake is inadequate
- Gastrointestinal complications (including constipation, bloating, reflux, gastroparesis and swallowing dysfunction) are monitored every 6 months
- Gastrointestinal complications (including constipation, bloating, reflux, gastroparesis and swallowing dysfunction) are actively treated and prevented (SCREENING)
- Annual discussion of gastrostomy tube feeding and timely intervention as part of usual care
- Enteral feeding via PEG or RIG should be available via an experienced team of anesthetists, gastroenterologists and interventional radiologists
- There is a re-feeding plan to prevent ‘re-feeding syndrome’ in cachectic patients

### Renal and bladder management

- Annual assessment of renal function (SCREENING) (by assessing full blood count (monitor for anemia), urea, electrolytes, cystatin C and urate (for patients on loop diuretic))
- Fluid intake is assessed yearly
- The multidisciplinary team should be able to access advice from a nephrologist to manage renal failure and an urologist to manage renal calculi

### Respiratory management

- Respiratory assessments are performed every 6–12 months in adult DMD patients (SCREENING) by experienced clinicians
- Adult DMD patients are referred to a home ventilation team if needed (SCREENING)
- Routine respiratory measurements should include forced vital capacity (FVC) (only if patients are not yet established on Bilevel Positive Airway Pressure, BiPAP) and peak cough flow (PCF) in adult DMD patients
- Overnight respiratory monitoring is used to investigate nocturnal hypoventilation to aid decision making (optimal timing of initiation of long term ventilation) at least once per year or more on indication
- Adult DMD patients are recommended to initiate lung volume recruitment (LVR) techniques with an LVR bag or Mechanical Insufflation-Exsufflation (MI-E) device (SCREENING) if they experience chest infections, have difficulty mobilizing secretions, or their PCF is < 270 l/min
- Adult DMD patients are offered secretion management including Lung Volume Recruitment (LVR) bags and Mechanical Insufflation-Exsufflation (MI-E) devices
- Referring for non-invasive ventilation is recommended in 1) asymptomatic patients whose FVC < 50% predicted, 2) in the presence of symptoms of nocturnal hypoventilation regardless of FVC (morning headaches, fatigue, weight loss, poor sleep quality), and 3) for patients with abnormal sleep studies
- Offering additional daytime non-invasive ventilation if patients develop symptoms of hypoventilation
- Adult DMD patients have a respiratory care plan for respiratory illness and emergencies
- Adults with DMD are recommended to receive up to date immunization according to the national guidelines (including influenza, covid and pneumococcal vaccines)

### Cardiac management

- Annual cardiologist follow-up with assessment of cardiac function (SCREENING)
- Annual assessment of cardiac function with echocardiography and/or electrocardiogram(ECG) (SCREENING)
- cMRI is considered at baseline to detect pre-clinical cardiac involvement and guide treatment
- Consider twenty-four hour ECG recording to examine heart rhythm
- Assessment of cardiac function with cMRI once every 2–5 years or on clinical indication
- Adult DMD patients are treated with ACEi (and a beta-blocker if needed) and/or ARBs (SCREENING)
- DMD patients are recommended to start ACEi before transition, but if not, all adult DMD patients should be recommended to start and be up-titrated to the maximum tolerated dose
- Beta-blockers are initiated in adult DMD with systolic dysfunction
- A mineralocorticoid inhibitor is offered to adult DMD patients with systolic dysfunction or if there is evidence of myocardial fibrosis
- Renal function is monitored regularly and there is attention to fluid intake (especially during intercurrent illness and if adding a loop of diuretic)

### Bone health management

- Adult DMD patients on long term steroid treatment are assessed annually for symptoms and signs of steroid induced osteoporosis with a lateral spine x-ray and DXA scan (lateral spine x-rays should be prioritized, and DXA scans require individualized plans with adjustments for height) (SCREENING)
- Consult with a bone health specialist at the earliest sign of a fracture
- Annual assessment of serum 250H vitamin D (should be maintained > 50 nmol/l) and dietary calcium intake
- A vitamin D supplement (at least 800-1000iu/day) and sufficient dietary calcium intake is recommended to all adult DMD patients
- Bone protective therapies such as bisphosphonates or comparable medications are considered to treat fragility fractures

### Orthopedic management

- Joint range of motion is assessed at least once every six months by one of the members of the multidisciplinary team (such as the physiotherapist) (SCREENING)
- Visual monitoring for scoliosis at least once every six months in non-ambulatory adults with DMD (SCREENING)
- In the late non-ambulatory stage, a spine X-ray is performed annually when there is any concern about progression in adults with a known scoliosis
- Consider posterior spine fusion during the late non-ambulatory stage for adults with a progressive curve in defined situations (consult with the respiratory physician and cardiologist to ensure that lung and heart function are sufficient to proceed with the surgical intervention)
- Consider surgery on foot and Achilles tendon in selected situations

### Participation, psychosocial and palliative care

- A mental health clinician (such as a psychologist or social worker) is part of the multidisciplinary team (SCREENING)
- Mental health and quality of life of adults with DMD and their families are assessed at every clinic (SCREENING)
- Adults with DMD and their families receive ongoing mental support (SCREENING)
- A clinical neuropsychologist is part of the multidisciplinary team, or is available to the team (SCREENING)
- Neuropsychological evaluations and interventions for learning, emotional and behavioral problems are provided (SCREENING)
- Needs for vocational support are assessed
- Age-appropriate independence and social development are encouraged
- A psychiatrist is available to the team (SCREENING)
- Anti-depressant therapy, anxiolytic and/or psychological therapy is considered where appropriate
- Adult DMD patients have the opportunity to discuss end of life issues
- Symptom management is provided to patients with more advanced disease
- Adult DMD patients have access to a palliative care consultant

### Transition

- There is close contact between the pediatric and adult health services to facilitate transition and transfer to adult health services (with the pediatric team being responsible for the transition process)
- Families of (young) adults with DMD might need guidance on guardianship or conservatorship

### Primary care

- The primary care provider is part of the care team and works in partnership with the multidisciplinary team

### Perioperative and anesthetic care

- Anesthetic care is preferably provided by centers with expertise in the care of DMD patients (this may not always be possible, for example in case of an emergency)
- In case of an emergency setting where transfer to the (expert) center is not possible, advice is given to the local hospital (involved) by the expert center (if the expert center knows that a patient is hospitalized in a local hospital; patients are educated by the expert center to inform professionals in local hospitals to get in contact with the expert centers)
- There is always a pre-anesthetic assessment which includes assessments of 1) airway (mouth opening), 2) cardiac status (echo/cMRI) and 3) respiratory status (SCREENING)
- There is close liaison between the surgical, anesthetic, cardiac and respiratory teams
- The anesthetists are aware that DMD patients are at risk of cardiac and respiratory decompensation and steroid induced adrenal insufficiency during and after surgery
- The anesthetists are aware of the risk of acute rhabdomyolysis when using halothanes

### Emergency management

- An emergency card is recommended for DMD patients
- In case of an emergency setting where transfer to the (expert) center is not possible, advice is given to the local hospital (involved) by the expert center (if the expert center knows that a patient is hospitalized in a local hospital; patients are educated by the expert center to inform professionals in local hospitals to get in contact with the expert centers)
- Adult DMD patients have a plan to manage steroid-induced adrenal insufficiency during acute illness

### Patient satisfaction

Monitoring patient satisfaction with health care services regularly

### References

Birnkrant et al. (2018). Diagnosis and management of Duchenne muscular dystrophy, part 1: diagnosis, and neuromuscular, rehabilitation, endocrine, and gastrointenstinal and nutritional management. Lancet Neurology; 17(3):251-267.

Birnkrant et al. (2018). Diagnosis and management of Duchenne muscular dystrophy, part 2: respiratory, cardiac, bone health, and orthopaedic management. Lancet Neurology; 17(4):347-361.

Birnkrant et al. (2018). Diagnosis and management of Duchenne muscular dystrophy, part 3: primary care, emergency management, psychosocial care, and transitions of care across lifespan. Lancet Neurology; 17(5): 445-455.

Narayan S et al. (2022). Adult North Star Network (ANSN): Consensus Document for Therapists Working with Adults with Duchenne Muscular Dystrophy (DMD) – Therapy guidelines. Journal of Neuromuscular Diseases; 9:365-381.

Quinlivan R et al. (2021). Adult North Star Network (ANSN): Consensus Guideline For The Standard Of Care Of Adults With Duchenne Muscular Dystrophy. Journal of Neuromuscular Diseases; 8:899-926.

## Abbreviations

ADC program: Accredited duchenne centers program
DMD: Duchenne muscular dystrophy
WDO: World duchenne organization
